# Editorial: Plant Immunity against Viruses

**DOI:** 10.3389/fmicb.2017.00520

**Published:** 2017-03-29

**Authors:** Jian-Zhong Liu, Feng Li, Yule Liu

**Affiliations:** ^1^College of Chemistry and Life Sciences, Zhejiang Normal UniversityJinhua, China; ^2^College of Horticultural and Forestry Sciences, Huazhong Agricultural UniversityWuhan, China; ^3^School of Life Sciences, Tsinghua UniversityBeijing, China

**Keywords:** immunity, plant viruses, gene silencing, disease resistance gene, genome editing

Plant viruses, the simple obligate intracellular parasites with small genomes, rely entirely on host machineries for their life cycle including replication, intracellular (cell-to-cell) and systemic movement (Nelson and Citovsky, 2005). Virus infections pose serious threats to agriculture and cause huge economic losses. Despite encoding only a limited number of proteins, numerous interactions of viral RNAs/proteins with host factors have puzzled the plant virologists for over a century and the complexity of these interactions is just becoming understood.

Plants have developed two major strategies to counteract virus infections: resistance (*R*) gene-mediated, and RNA silencing-based defenses. In addition, the mutation in essential genes for viral infection also causes plant resistance against viruses, called recessive gene-mediated resistance. These approaches have been used in crop protections and have shown significant economic impact (Abel et al., 1986; Whitham et al., 1996; Baulcombe, 2004; Kang et al., 2005; Wang and Krishnaswamy, 2012).

This Research Topic combines 13 publications, including 9 review articles and 4 research articles, covering almost every aspect of plant-virus interactions. The featured in-depth topic reviews in various sub-fields provide readers a convenient way to understand the current status of the related sub-fields and the featured research articles expand the current knowledge in related sub-fields.

Not unexpectedly, vast majority of the papers in this Research Topic are related to gene silencing but with totally distinct emphasis. Khalid et al. summarizes the applications of various small RNA based genetic engineering (SRGE) in crop protection, focusing on the technology evolution and successful cases in different crops. Andika et al. reviews the current information on the molecular aspects of antiviral RNA silencing in roots, with emphasis on the interactions between host antiviral defense and soil-borne viruses. The distinctive characteristic features of RNA silencing in roots relative to shoots are summarized. Moon and Park review how the RNA silencing pathway cross-talks with the resistance (*R*) gene-mediated defense. Several components involved in host RNA silencing mechanisms have recently been shown to be required for *R* gene-mediated defense. It seems that it is a common phenomenon that miRNAs or siRNAs regulate *R*-gene mediated resistance through targeting *R* genes for cleavage in plants (Moon and Park). It is plausible that the cross-talk between these two defense pathways is to maximize the efficiency of defense responses against viral infections (Nakahara and Masuta, 2014). Huang et al. summarize the various scenarios of host- and pathogen-derived sRNAs or pathogen-induced host sRNAs in regulating host resistance/susceptibility or pathogen virulence/pathogenicity.

The zigzag model (Jones and Dangl, 2006) presents a classic view of the interactions between plants and non-viral pathogens. Ding (2010) considered dsRNA as the Pathogen-associated Molecular Patterns (PAMPs) of viral pathogen and RNA silencing (or RNAi) as a form of PTI against viruses. Recently, Mandadi and Scholthof (2013) further integrated these concepts into current plant-virus interaction models. In this model, dsRNAs produced during virus infection are regarded as viral PAMP; RNAi-mediated antiviral defense is analogous to PAMP-triggered immunity (PTI); viral suppressors of RNAi (VSR) such as coat proteins (CPs), movement proteins (MPs), and replicase are regarded as avirulent (Avr) factors or effectors; *R* gene-mediated viral resistance is considered as viral effector-triggered immunity (ETI). Moon and Park and Gouveia et al. review how this model is shaped in details. In addition, Gouveia et al. reviewed the recent progresses in antiviral immune receptors and co-receptors involved in antiviral innate immunity in plants and describe the NIK1-mediated antiviral signaling, which is specific to plant DNA viruses and relies on transmembrane receptor-mediated translational suppression for defense. It remains to be seen whether this is an exception or a common viral defense mechanism used by plants.

Recessive resistance is conferred either by a recessive gene mutation that encodes a host factor critical for viral infection or by a mutation in a negative regulator of plant defense responses, possibly due to the autoactivation of defense signaling. Eukaryotic translation initiation factor (eIF) 4E and eIF4G and their isoforms are the most widely exploited recessive resistance genes in several crop species (Kang et al., 2005). Hashimoto et al. thoroughly review the recent advances in recessive resistance studies not just limited to eIF4E and eIF4G.

The disturbance of chloroplast components and functions is largely responsible for the chlorosis symptoms that are associated with virus infection. Chloroplast is not only the organelle that conducts photosynthesis but also the site for the biosynthesis of SA and JA, two major phytohormones that play roles in disease and resistance. Zhao et al. review the different aspects of chloroplast during plant-virus interactions, particularly focusing on the interactions between chloroplast and viral proteins that underlie the interplay between chloroplast and virus. Liu et al. review the major advances that have been made recently in identifying both the virulence/avirulence factors of *Soybean mosaic virus* (SMV) and mapping of SMV resistant genes in soybean. A special focus is given to the progress made in dissecting the SMV resistant signaling pathways using virus-induced gene silencing (VIGS). Romay and Bragard review the latest progress in plant antiviral defenses mediated by genome editing systems (GES). TALEN and CRISPR-Cas9 have been applied to generate resistance against plant viruses in the families of *Geminiviridae* and *Potyviridae*. Interestingly, the newly developed CRISPR-Cas systems using new versions of Cas9 proteins, capable to cleave ssRNA molecules, can be applied to target RNA viruses (Sampson et al., 2013; Abudayyeh et al., 2016). One advantage of genome editing is that the transgenes can be removed via segregation after editing. Some genome-edited crops have already been made available without being restricted by the US Department of Agriculture (Waltz, 2016). The other advantage of genome editing is that multiple alleles or genes can be edited simultaneously.

Application of next generation sequencing (NGS) technology in small RNA profiling has significantly impact on the studies of plant-virus interactions. Li et al. investigate profiles of the Cucumber green mottle mosaic virus (CGMMV)-derived siRNAs (vsiRNA) in infected leaves and fruits of *L. siceraria* using NGS. The vsiRNA patterns of abundance, origination and polarity, hotspot distribution, GC content and 5′ terminal nucleotide are compared between the infected leaves and fruits. The similarities and distinct differences are revealed by this analysis. Co-infection of none-coding satellite RNAs (sat-RNAs) usually inhibits replication and attenuates disease symptoms of helper viruses. However, Xu et al. reveal that co-infection of none-coding satellite RNAs (sat-RNAs) of Beet black scorch virus (BBSV) enhances the replication and the symptoms of BBSV on *N. benthamiana* possibly through competitively occupying or saturating host silencing machinery. Fang et al. narrow down the functional domains of 2b protein of Cucumber mosaic virus (CMV) for dsRNA binding and Argonaute (AGO) interaction. Their findings demonstrate that the dsRNA-binding activity of the 2b is essential for virulence, whereas the 2b-AGO interaction is necessary for interference with RDR1/6-dependent antiviral silencing in Arabidopsis. Alpha-momorcharin (α-MMC) is a type-I ribosome inactivating protein (RIP) in *Momordica charantia*. Yang et al. provide evidence that α-MMC plays a positive role in the resistance against CMV in *M. charantia* and the antiviral activities of α-MMC may be achieved through up-regulating JA and ROS signaling pathway.

We hope that this Research Topic helps readers to have a better understanding of the progresses that have been made recently in plant immunity against viruses. A deeper understanding of plant antiviral immunity will facilitate the development of innovative approaches for crop protections and improvements.

## Author contributions

All authors listed, have made substantial, direct and intellectual contribution to the work, and approved it for publication.

### Conflict of interest statement

The authors declare that the research was conducted in the absence of any commercial or financial relationships that could be construed as a potential conflict of interest.
